# Analysis of risk factors for acute kidney injury in fulminant myocarditis and development of a renal prognosis prediction model

**DOI:** 10.3389/fcvm.2026.1796802

**Published:** 2026-04-01

**Authors:** Changli Sun, Shuai Huo, Pei Yu

**Affiliations:** 1 NHC Key Lab of Hormones and Development and Tianjin Key Lab of Metabolic Diseases, Tianjin Medical University Chu Hsien-I Memorial Hospital & Institute of Endocrinology, Tianjin, China; 2 Department of Nephrology & Blood Purification Center, The Second Hospital of Tianjin Medical University, Tianjin, China; 3 Department of Nephrology, Central China Fuwai Hospital, Zhengzhou, Henan, China

**Keywords:** acute kidney injury, chronic kidney disease, fulminant myocarditis, prediction model, prognosis

## Abstract

**Objective:**

This observational cohort study aimed to identify risk factors for acute kidney injury (AKI) related to fulminant myocarditis (FM) and for the progression of AKI to chronic kidney disease (CKD), as well as to develop a risk prediction model to help improve the renal prognosis in FM patients.

**Methods:**

Clinical data were collected for FM patients treated at Central China Fuwai Hospital between December 1, 2018 and June 30, 2025. Patients were categorized into AKI and non-AKI groups, and surviving AKI patients were followed for at least 3 months to observe CKD progression. The logistic regression model was used to analyze the risk factors for FM-associated AKI and its progression to CKD. A receiver operator characteristic (ROC) curve was drawn to evaluate the performance of the clinical risk factor model.

**Results:**

Of the 408 FM patients included in this study, 201 (49.2%) exhibited FM-associated AKI. Male gender, elevated baseline N-terminal pro b-type natriuretic peptide (NT-pro BNP) and procalcitonin levels and reduced left ventricular ejection fraction were identified as risk factors for FM-associated AKI (*P* < 0.05). The clinical risk prediction model including the above factors showed excellent performance for predicting FM-associated AKI (area under curve [AUC] = 0.917, 95% confidence interval [CI]: 0.875–0.960, *P* < 0.001). Of 188 patients who survived to discharge in the FM-associated AKI group, 66 (35.1%) progressed to CKD. Independent risk factors for progression to CKD included delayed treatment and elevated serum creatinine at discharge. The clinical risk prediction model including these factors exhibited excellent performance for predicting FM-associated AKI to CKD (AUC = 0.898, 95% CI: 0.817–0.979, *P* < 0.001).

**Conclusion:**

FM patients have a high risk of renal complications. Patients with FM-associated AKI are also at risk for developing CKD. A logistic prediction model based on independent risk factors can predict poor renal prognosis in FM-associated AKI patients, but this prediction model represents a preliminary exploration and requires external validation for further clinical application.

## 1 Introduction

Fulminant myocarditis (FM) is a systemic disease characterized by inflammatory damage to the myocardium, which can be caused by infection, autoimmune disease, and other etiologies. According to the Chinese expert consensus statement (1), the diagnostic criteria for FM includes: sudden onset with obvious pre-viral symptoms, especially weakness and loss of appetite, followed by rapid development of severe hemodynamic disorders. Laboratory tests show severe damage to the myocardium, and echocardiography reveals diffuse weakened ventricular wall movement. While FM accounts for only 10%–15% of all myocarditis cases (2), it represents a dangerous condition as its rapid onset and progression can result in significant hemodynamic instability, cardiogenic shock, malignant arrhythmias, and even systemic multi-organ failure. The mortality rate during the early stages of FM is alarmingly high, ranging from 50% to 75% (3–5). The hemodynamic disturbances and hypoxemia associated with FM can compromise renal blood perfusion, potentially leading to acute kidney injury (AKI) (6). Furthermore, immune-mediated damage and stress-induced injury, facilitated by the release of inflammatory mediators and cytokines, can exacerbate renal impairment (7, 8). Extracorporeal membrane oxygenation (ECMO) is the preferred treatment modality for FM. However, ECMO-associated complications, such as ischemia–reperfusion injury, hemolysis and hyperlactatemia, may increase the risk of renal complications or worsen existing renal conditions (9, 10). Therefore, multiple factors can contribute to renal damage during the course of FM. The reported morbidity of FM-associated AKI is as high as 59% (11). The occurrence of AKI substantially elevates the risk of developing chronic kidney disease (CKD) and end stage renal disease (ESRD), thereby potentially impacting patients' long-term survival (12). Consequently, the identification of risk factors for FM-associated AKI and its progression to CKD is crucial for predicting the trajectory of AKI and mitigating the risks of poor renal prognosis and mortality. Such research to determine the factors associated with the adverse renal prognosis of FM patients is lacking. Hence, we conducted this retrospective, observational cohort study with the objectives of developing a clinical risk prediction model for early identification of high-risk patients and providing a foundation for reducing the risk of a poor renal prognosis associated with FM.

## 2 Materials and methods

### 2.1 Study population

Patients with FM who were treated at the Central China Fuwai Hospital between December 1, 2018 and June 30, 2025 were included according to the following inclusion criteria: (1) age 18 years or older; (2) meeting the diagnostic criteria for FM (1); (3) no prior history of CKD confirmed by medical records, repeated measurement of an estimated glomerular ﬁltration rate (eGFR) < 60 mL·min^−1^·1.73 m^−2^ for ≥3 months, or kidney replacement therapy before admission. The exclusion criteria for this study were: (1) AKI occurring in patients with pre-existing CKD; (2) complications including urinary tract obstruction and infection, malignant tumors, or pregnancy; (3) history of kidney transplantation; and (4) incomplete clinical data or loss of follow-up. The study protocol was approved by the Ethics Committee of Central China Fuwai Hospital (Approval Number: 2025-101) and was conducted in accordance with the Declaration of Helsinki. The patients provided their written informed consent to participate in this study.

### 2.2 Diagnostic criteria and staging of AKI

The criteria used for AKI diagnosis and staging were based on the 2012 Kidney Disease Improving Global Outcomes (KDIGO) guidelines (13). Briefly, AKI was diagnosed if any of the following conditions were met: increase in serum creatinine by ≥26.5 μmol/L (0.3 mg/dL) within 48 h; increase in serum creatinine to >1.5 times the baseline value within 7 days; or urine output <0.5 mL/kg/h for more than 6 h. AKI was staged as follows: Stage 1: increase in serum creatinine to 1.5–1.9 times the baseline values or by ≥26.5 μmol/L (0.3 mg/dL), or urine output <0.5 mL/kg/h for 6–12 h. Stage 2: increase in serum creatinine to 2.0–2.9 times the baseline value, or urine output <0.5 mL/kg/h for more than 12 h. Stage 3: increase in serum creatinine to >3 times the baseline or by ≥353.6 μmol/L (4.0 mg/dL), performance of renal replacement therapy, urine output <0.3 mL/kg/h for more than 24 h, or no urine for >12 h.

The included patients were categorized into an AKI group and a non-AKI group. The median time from admission to the diagnosis of FM-associated AKI was 48 h. Considering the difficulty of accurately measuring urine output and that urine output was greatly influenced by many factors, urine output was not used as a diagnostic indicator for AKI in this study (14). Surviving FM-associated AKI patients were followed up for at least 3 months and further divided into a CKD group and a non-CKD group according to the progression to CKD.

### 2.3 Data collection

Baseline characteristics were collected, including demographic information, medical history, time interval from disease onset to treatment, presence and site of infection, New York Heart Association (NYHA) classification of cardiac function, and medication status [including diuretics, angiotensin-converting enzyme inhibitors (ACEI), angiotensin receptor blockers (ARBs), and beta-blockers]. Additionally, data on the utilization of mechanical circulatory support [such as ECMO, intra-aortic ballon pump (IABP), and continuous renal replacement therapy (CRRT)], blood pressure, baseline laboratory indicators [including white blood cell count, red cell distribution width, hemoglobin, C-reactive protein, blood urea nitrogen, serum creatinine, retinol-binding protein, cystatin-C, albumin, low-density lipoprotein, N-terminal pro b-type natriuretic peptide (NT-pro BNP), creatine kinase-MB, procalcitonin, and left ventricular ejection fraction] were obtained, and eGFR was calculated based on the serum creatinine levels using the Chronic Kidney Disease Epidemiology Collaboration (CKD-EPI) equation (15). FM-associated AKI patients who survived to discharge were followed up for at least 3 months after discharge, during which serum creatinine levels were measured.

### 2.4 Study endpoint

The primary study endpoint was progression of FM-associated AKI to CKD. According to the KDIGO 2012 AKI diagnosis and treatment guidelines (13), the transition from AKI to CKD was deﬁned as eGFR < 60 mL·min^−1^·1.73 m^−2^ for ≥3 months post-AKI onset.

### 2.5 Statistical analysis

All statistical analyses in this study were conducted using SPSS 25.0. Normality of continuous data was tested using the Shapiro–Wilk test and Q-Q plot, and data with *P* > 0.05 in the Shapiro–Wilk test were considered normally distributed. Normally distributed data were presented as mean ± standard deviations $\left(\bar{x}\pm s\right)$, whereas non-normally distributed data were presented as median (interquartile range). Categorical variables were presented as count (percentage). Comparisons between groups were conducted using the Student's *t* test and Mann–Whitney *U* test for continuous variables and the chi-square test for categorical variables. Logistic regression analysis was performed to identify inﬂuencing factors for FM-associated AKI and its progression to CKD. Candidate predictors for logistic regression model were selected based on clinical relevance and univariable analysis results (*P* < 0.05). Goodness of fit was verified by the Hosmer-Lemeshow test, and *P* > 0.05 was regarded as indicative of an acceptable model. Receiver operating characteristic (ROC) curve analysis and area under the curve (AUC) calculation were applied to assess the performance of the prediction model, with *P* < 0.05 indicating statistical significance.

## 3 Results

### 3.1 Study population

**Figure 1:**
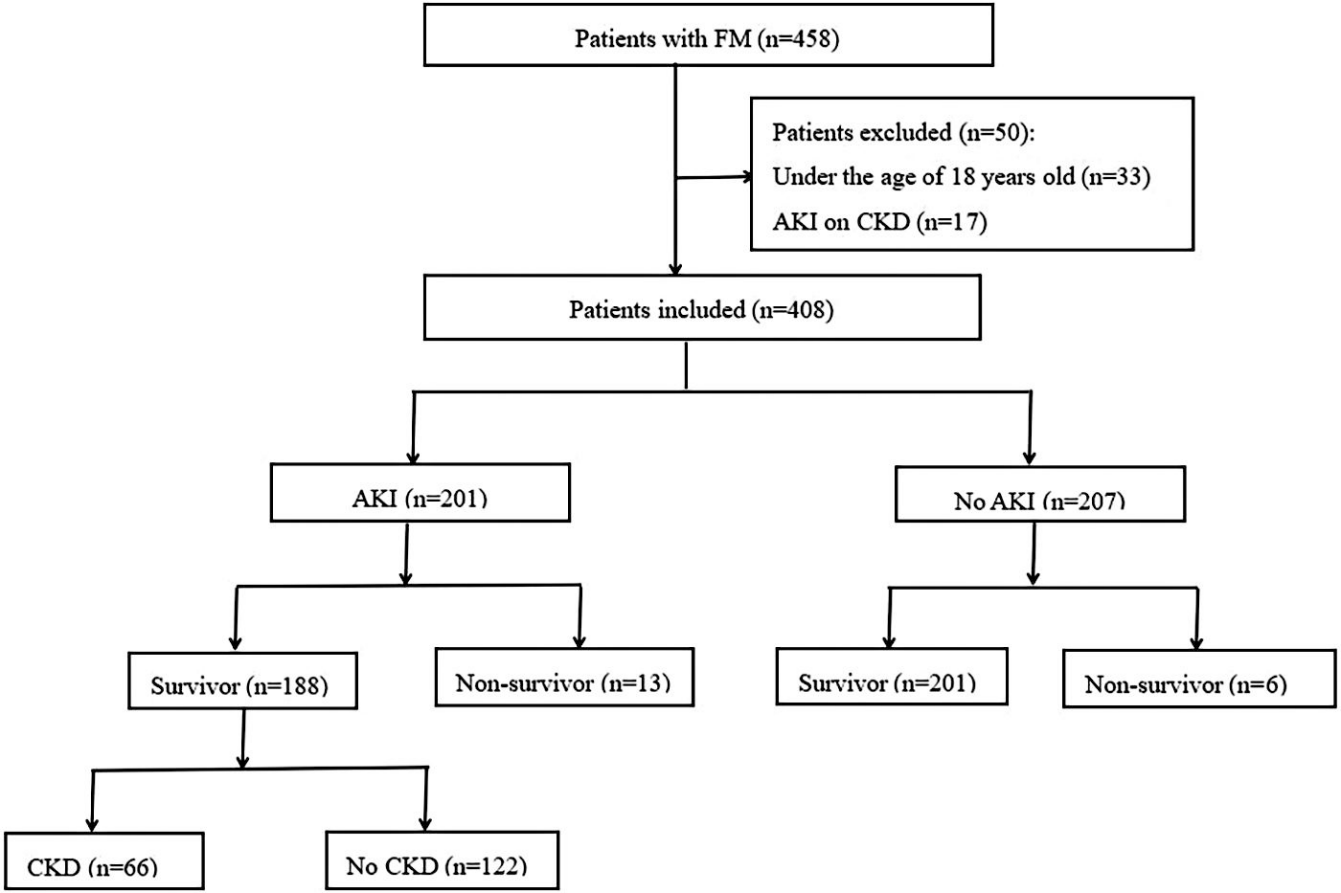
Flowchart of inclusion of patients with FM.

A total of 458 patients diagnosed with FM were initially enrolled in this study. We excluded 33 patients who were less than 18 years of age and 17 patients who were experiencing AKI based on CKD. Consequently, 408 adult patients were included in the final analysis, consisting of 200 males and 208 females. Among these 408 patients, 201 (49.2%) developed FM-associated AKI. Overall, 188 of the AKI patients survived to discharge, with an in-hospital mortality rate of 6.4%. Of the surviving AKI patients, 66 (35.1%) experienced subsequent progression to CKD. A flow diagram of patient selection is presented in Figure 1.

### 3.2 Comparison of clinical data between AKI and non-AKI groups of FM patients

Compared with the non-AKI group, the AKI group exhibited significantly higher age and a greater proportion of males. Additionally, the AKI group had a higher prevalence of NYHA grade IV heart failure and increased utilization of ECMO and IABP. Furthermore, the AKI group demonstrated higher baseline values for white blood cell count, red cell distribution width, C-reactive protein, blood urea nitrogen, serum creatinine, cystatin-C, NT-pro BNP, creatine kinase-MB and procalcitonin. Conversely, the AKI group had a lower proportion of beta-blocker use as well as lower baseline values for eGFR, albumin, and left ventricular ejection fraction (Table 1).

**Table 1:** Comparison of baseline clinical characteristics of patients in the AKI group and non-AKI group.

Variables	AKI (*n* = 201)	Non-AKI (*n* = 207)	*T*/*z*/*χ*^2^	*P-*value
Male gender	118 (58.7%)	82 (39.6%)	5.905	0.015
Age (years)	50 (36, 59)	38 (28, 49)	3.502	<0.001
Time interval from onset to treatment (h)	12 (5, 24)	24 (7, 48)	1.487	0.137
Comorbidities				
Diabetes mellitus	24 (11.9%)	16 (7.7%)	0.952	0.329
Hypertension	41 (20.3%)	34 (16.4%)	0.486	0.486
Coronary heart disease	73 (36.3%)	67 (32.3%)	0.240	0.624
Infection				
Lung infection	161 (80.1%)	145 (70.0%)	6.245	0.182
Lung and pancreas infection	5 (2.5%)	0 (0%)		
Alimentary infection	3 (1.5%)	5 (2.5%)		
NYHA grade				
Class II	5 (2.5%)	23 (11.1%)	3.248	0.001
Class III	26 (12.9%)	54 (26.0%)		
Class IV	170 (84.6%)	130 (62.9%)		
Medication				
Diuretics	169 (84.0%)	181 (87.4%)	0.336	0.562
ACEI or ARB	10 (4.9%)	16 (7.7%)	1.973	0.160
Beta-blockers	97 (48.2%)	137 (66.1%)	5.421	0.020
ECMO	145 (72.2%)	77 (37.1%)	19.938	<0.001
IABP	138 (68.6%)	77 (37.1%)	15.908	<0.001
Systolic pressure (mmHg)	103 ± 23	107 ± 19	1.300	0.195
Diastolic pressure (mmHg)	66 (55, 76)	65 (58, 79)	1.089	0.276
White blood cell count (×10^9^/L)	12.3 (8.9, 16.5)	10.3 (7.2, 13.4)	2.613	0.009
Red cell distribution width (%)	42.3 (40.2, 43.8)	40.7 (39.6, 43.0)	1.970	0.049
Hemoglobin (g/L)	123 (106, 142)	122 (113, 140)	0.442	0.659
C-reactive protein (mg/L)	49.0 (14.6, 85.5)	15.7 (6.4, 54.2)	3.003	0.003
Blood urea nitrogen (mmol/L)	9.5 (6.5, 13.4)	5.7 (4.2, 7.8)	5.697	<0.001
Serum creatinine (µmol/L)	116 (81, 167)	61 (49, 75)	7.877	<0.001
eGFR (mL·min^−1^·1.73 m^−2^)	56.8 (40.3, 80.4)	100.2 (83.9, 115.0)	7.379	<0.001
Retinol-binding protein (mg/L)	27.4 (20.3, 33.6)	27.4 (18.1, 35.2)	0.066	0.947
Cystatin-C (mg/L)	1.4 (1.0, 1.9)	0.9 (0.8, 1.3)	5.270	<0.001
Albumin (g/L)	34.9 ± 5.1	36.9 ± 4.9	2.597	0.010
Low-density lipoprotein (mmol/L)	1.8 (1.3, 2.5)	1.9 (1.4, 2.7)	0.808	0.419
NT-pro BNP (pg/mL)	4,212 (1,368, 8,320)	680.5 (342.0, 2,347.7)	6.828	<0.001
Creatine kinase-MB (U/L)	80 (34, 188)	49 (23, 88.5)	2.792	0.005
Procalcitonin (ng/mL)	2.6 (0.6, 7.8)	0.18 (0.08, 1.01)	5.853	<0.001
Left ventricular ejection fraction (%)	27 (19, 36)	39 (28, 53)	4.719	<0.001

AKI, acute kidney injury; NYHA, New York Heart Association; ACEI, angiotensin-converting enzyme inhibitors; ARBs, angiotensin receptor blockers; ECMO, extracorporeal membrane oxygenation; IABP, intra-aortic ballon pump; eGFR, estimated glomerular filtration rate; NT-pro BNP, N-terminal pro B-type natriuretic peptide. Normally distributed data are presented as mean ± standard deviation $\left(\bar{x}\pm s\right)$, non-normally distributed data as median (Q1–Q3), and categorical data as *n* (%).

### 3.3 Risk factors for FM-associated AKI

Univariable analysis identified several potential risk factors for FM-associated AKI, including male gender; age; beta-blocker use; elevated baseline levels of C-reactive protein, NT-pro BNP, creatine kinase-MB, and procalcitonin; as well as reduced albumin and left ventricular ejection fraction. With the above-mentioned factors incorporated into the multivariate logistic regression analysis, the results showed that male gender, elevated NT-pro BNP and procalcitonin levels, and reduced left ventricular ejection fraction were independent risk factors for FM-associated AKI (Table 2).

**Table 2:** Logistic regression analyses of potential risk factors for FM-associated AKI.

Variables	Univariable analysis		Multivariable analysis	
	OR (95% CI)	*P*-value	OR (95% CI)	*P*-value
Male gender	2.162 (1.156, 4.041)	0.016	5.160 (1.790, 14.873)	0.002
Age (years)	1.038 (1.016, 1.061)	0.001	1.028 (0.997, 1.060)	0.082
Beta-blockers	2.110 (1.121, 3.972)	0.021	1.532 (0.600, 3.910)	0.372
C-reactive protein (mg/L)	1.008 (1.002, 1.013)	0.012	1.005 (0.998, 1.013)	0.185
Albumin (g/L)	0.920 (0.863, 0.982)	0.013	0.980 (0.891, 1.079)	0.684
NT-pro BNP (pg/mL)	1.000 (1.000, 1.001)	<0.001	1.000 (1.000, 1.001)	<0.001
Creatine kinase-MB (U/L)	1.005 (1.002, 1.009)	0.002	1.006 (1.000, 1.011)	0.056
Procalcitonin (ng/mL)	1.281 (1.131, 1.451)	<0.001	1.232 (1.052, 1.444)	0.010
Left ventricular ejection fraction (%)	0.917 (0.889, 0.945)	<0.001	0.937 (0.899, 0.976)	0.002

eGFR, estimated glomerular filtration rate; NT-pro BNP, N-terminal pro B-type natriuretic peptide.

### 3.4 Risk prediction model for FM-associated AKI

**Figure 2:**
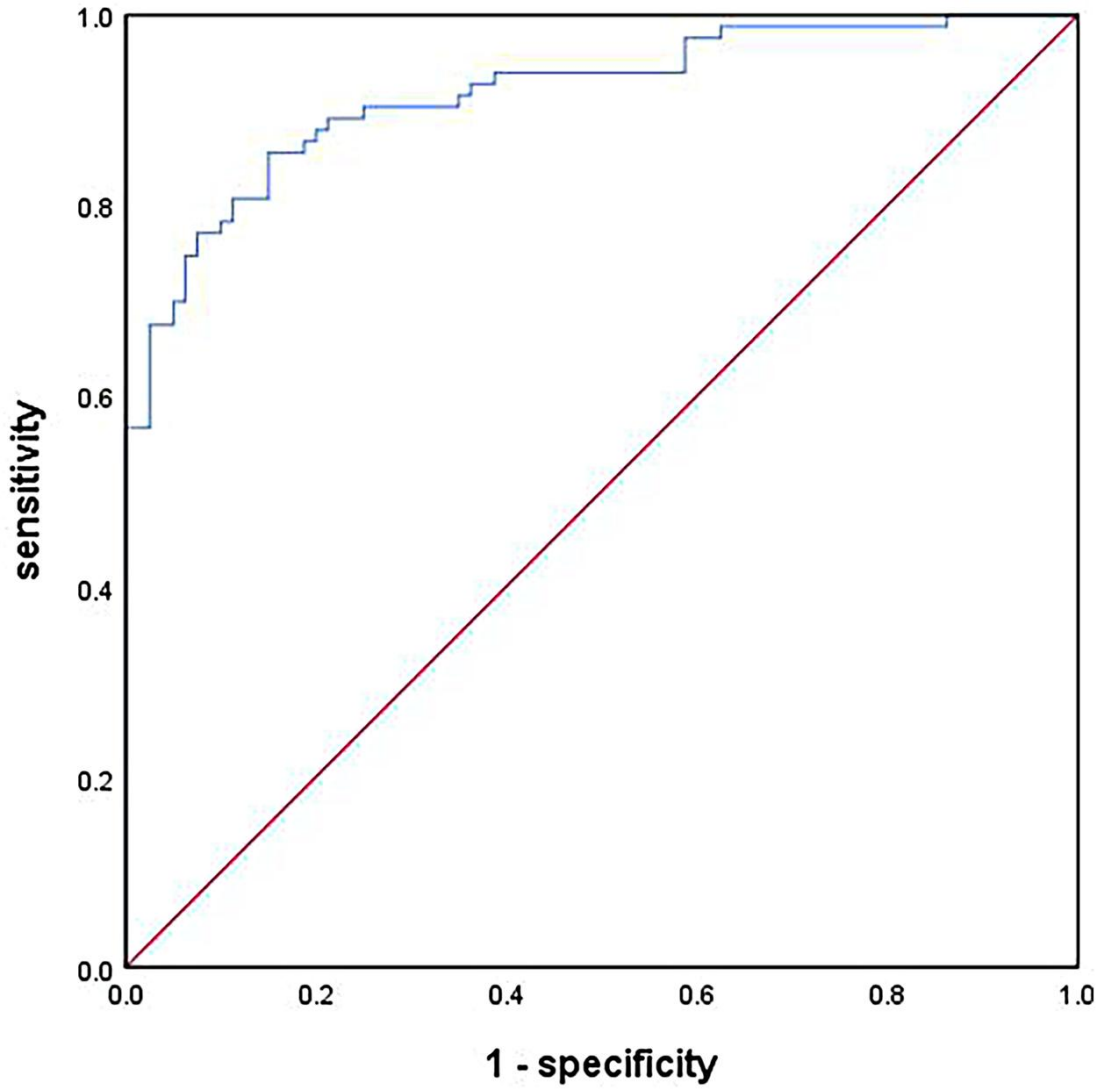
Performance of clinical model for predicting FM-associated AKI.

We used the independent risk factors associated with FM-associated AKI on the multivariate regression analysis to establish a clinical risk prediction model. The variance inflation factors for all the variables in the model were less than 2, suggesting that the multicollinearity among the variables was small. The Hosmer–Lemeshow test (*χ*^2^ = 3.732, *P* = 0.880) and Nagelkerke pseudo *R*^2^ (*R*^2^ = 0.650) test results showed that the model had a good goodness of fit. On ROC curve analysis, this model exhibited excellent predictive performance for FM-associated AKI with an AUC of 0.917 (95% CI: 0.875–0.960, *P* < 0.001), and the sensitivity and specificity were 85.5% and 85.0% (Figure 2).

### 3.5 Comparison of clinical data between patients with progression of FM-associated AKI to CKD and those without progression to CKD

Among the 188 patients in the AKI group who survived to discharge, 66 (35.1%) experienced progression to CKD. Compared with the non-CKD group, the CKD group had higher proportions of males and a longer interval from onset to treatment. Additionally, the CKD group demonstrated higher baseline blood urea nitrogen and albumin levels, higher serum creatinine levels at discharge and 3 months, and lower eGFR values at baseline, discharge, and 3 months (Table 3).

**Table 3:** Comparison of baseline clinical characteristics of patients in CKD group and non-CKD group.

Variables	CKD (*n* = 66)	Non-CKD (*n* = 122)	*t*/*Z*/*χ*^2^	*P-*value
Male gender	51 (77.2%)	57 (46.7%)	5.869	0.015
Age (years)	51.7 ± 14.2	45.1 ± 17.3	1.657	0.102
Time interval from onset to treatment (h)	24 (6, 48)	10 (5, 24)	2.519	0.012
Comorbidities				
Diabetes mellitus	9 (13.6%)	7 (5.7%)	1.041	0.307
Hypertension	18 (27.2%)	18 (14.7%)	1.409	0.235
Coronary heart disease	29 (43.9%)	40 (32.7%)	0.820	0.365
Infection				
Lung infection	52 (78.9%)	104 (85.3%)	2.342	0.505
Lung and pancreas infection	5 (7.5%)	0 (0%)		
NYHA grade				
Class II	4 (6.0%)	0 (0%)	4.564	0.102
Class III	4 (6.0%)	25 (20.4%)		
Class IV	58 (88.0%)	97 (79.6%)		
Medication				
Diuretics	59 (89.3%)	114 (94.1%)	0.375	0.540
ACEI or ARBs	5 (7.5%)	7 (5.7%)	1.151	0.283
Beta-blockers	33 (50%)	79 (64.7%)	1.413	0.235
AKI stage				
Stage 1	15 (22.8%)	57 (46.7%)		
Stage 2	9 (13.6%)	18 (14.8%)	4.526	0.104
Stage 3	42 (63.6%)	47 (38.5%)		
CRRT	40 (60.6%)	47 (38.5%)	3.023	0.082
ECMO	44 (66.7%)	39 (31.9%)	0.820	0.365
IABP	46 (69.6%)	82 (67.2%)	0.041	0.839
Systolic pressure (mmHg)	103 (95, 111)	103 (89, 120)	0.269	0.788
Diastolic pressure (mmHg)	68 (56, 78)	65 (57, 71)	0.808	0.419
White blood cell count (×10^9^/L)	11.4 (8.8, 15.6)	11.7 (8.9, 16.6)	0.094	0.925
Red cell distribution width (%)	42.4 ± 4.0	41.6 ± 2.7	0.940	0.351
Hemoglobin (g/L)	122 ± 20	126 ± 20	0.782	0.437
C-reactive protein (mg/L)	58.9 (15.3, 106.6)	45.9 (11.6, 8.2)	1.278	0.201
Blood urea nitrogen (mmol/L)	11.4 ± 6.0	8.7 ± 4.0	2.098	0.040
Serum creatinine at baseline (µmol/L)	111 (94, 179)	113 (75, 129)	1.177	0.239
Serum creatinine at discharge (µmol/L)	172 (92, 242)	68 (54, 85)	4.999	<0.001
Serum creatinine at 3 months (µmol/L)	218 (140, 336)	72 (59, 81)	6.863	<0.001
eGFR at baseline (mL·min^−1^·1.73 m^−2^)	50.4 ± 25.0	71.5 ± 27.9	3.173	0.002
eGFR at discharge (mL·min^−1^·1.73 m^−2^)	50.7 ± 31.3	95.1 ± 28.5	5.920	<0.001
eGFR at 3 months (mL·min^−1^·1.73 m^−2^)	33.8 ± 19.1	100.6 ± 21.9	12.863	<0.001
Retinol-binding protein (mg/L)	32.2 (23.6, 36.5)	25.5 (19.2, 31.2)	1.695	0.090
Cystatin-C (mg/L)	1.1 (0.9, 1.8)	1.0 (0.8, 1.3)	1.709	0.087
Albumin (g/L)	36.0 ± 4.3	33.6 ± 4.8	2.146	0.036
Low-density lipoprotein (mmol/L)	2.0 ± 0.9	2.0 ± 1.3	0.111	0.912
NT-pro BNP (pg/mL)	3,321.0 (1,414.5, 6,968.5)	3,115.0 (1,146.1, 8,752.0)	0.256	0.798
Creatine kinase-MB (U/L)	83.0 (29.2, 134.5)	65.0 (26.7, 178.7)	0.215	0.830
Procalcitonin (ng/mL)	3.7 (1.4, 9.9)	1.6 (0.3, 6.4)	1.534	0.125
Left ventricular ejection fraction (%)	30 ± 13	32 ± 15	0.684	0.496

CKD, chronic kidney disease; NYHA, New York Heart Association; ACEI, angiotensin-converting enzyme inhibitors; ARBs, angiotensin receptor blockers; CRRT, continuous renal replacement therapy; ECMO, extracorporeal membrane oxygenation; IABP, intra-aortic ballon pumpe; eGFR, estimated glomerular filtration rate; NT-pro BNP, N-terminal pro B-type natriuretic peptide. Normally distributed data are presented as mean ± standard deviation $\left(\bar{x}\pm s\right)$, non-normally distributed data as median (Q1–Q3), and categorical data as *n* (%).

### 3.6 Risk factors for progression of FM-associated AKI to CKD

Univariable analysis identified potential risk factors for the progression of FM-associated AKI to CKD, including male gender, prolonged time interval from onset to treatment, AKI stage 3, and elevated levels of albumin at baseline and serum creatinine at discharge. Multivariable logistic regression analysis further demonstrated that prolonged time interval from onset to treatment and elevated serum creatinine at discharge were independent risk factors for the progression of FM-associated AKI to CKD (Table 4).

**Table 4:** Logistic regression analysis of potential risk factors for progression of FM-associated AKI to CKD.

Variables	Univariable analysis		Multivariable analysis	
	OR (95% CI)	*P*-value	OR (95% CI)	*P*-value
Male gender	3.696 (1.254, 10.899)	0.018	1.747 (0.334, 9.136)	0.508
Time interval from onset to treatment (h)	1.043 (1.010, 1.076)	0.009	1.049 (1.006, 1.095)	0.026
AKI stage	3.341 (1.075, 10.386)	0.037	0.979 (0.392, 2.447)	0.964
Serum creatinine at discharge (µmol/L)	1.028 (1.012, 1.045)	<0.001	1.030 (1.010, 1.050)	0.004
Albumin (g/L)	1.130 (1.004, 1.272)	0.042	1.099 (0.919, 1.314)	0.300

AKI, acute kidney injury; eGFR, estimated glomerular filtration rate.

### 3.7 Risk prediction model for progression of FM-associated AKI to CKD

**Figure 3:**
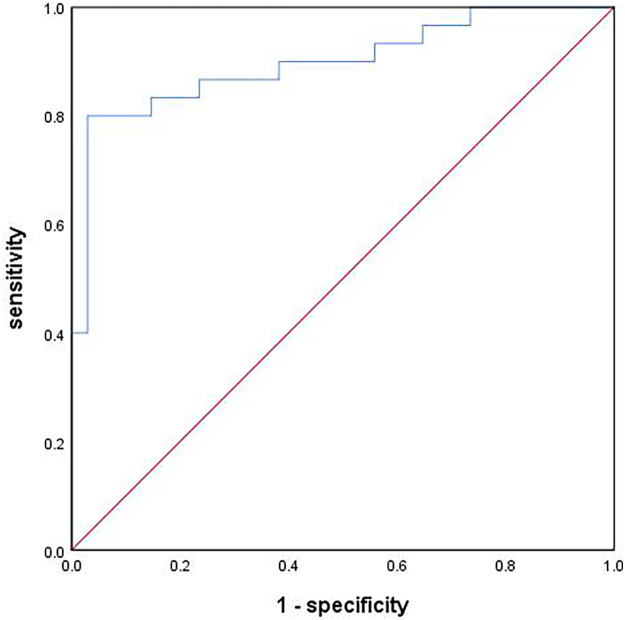
Performance of clinical model for predicting FM-associated AKI progression to CKD.

Based on these multivariable logistic regression analysis findings, the logistic predictive model for the progression of FM-associated AKI to CKD was developed. The variance inflation factors for all the variables in the model were less than 2, suggesting that the multicollinearity among the variables was small. The Hosmer–Lemeshow test (*χ*^2^ = 6.796, *P* = 0.559) and Nagelkerke pseudo *R*^2^ (*R*^2^ = 0.620) test results showed that the model had a good goodness of fit. The AUC value for the predictive ability of the model was 0.898 (95% CI: 0.817–0.979, *P* < 0.001), indicating excellent predictive performance, and the model had a sensitivity of 80.0% and a specificity of 97.1% (Figure 3).

## 4 Discussion

The hemodynamic disturbances, inflammatory storm, and treatment factors associated with FM significantly raise the risk of kidney injury. In our study, the incidence of FM-associated AKI was 49.2%, which was consistent with the results of Hao et al. (16) and slightly lower than the 59% reported by Yang et al. (11), which may be due to differences in study populations and FM severity. We identified male gender, elevated baseline NT-pro BNP and procalcitonin levels, and reduced left ventricular ejection fraction as risk factors for FM-associated AKI and included these factors in a predictive model, which exhibited excellent predictive performance.

Whether gender is an influencing factor for AKI is currently a subject of controversy. A large-scale prospective cohort study by Jiang et al. (17) indicated that male gender is an independent risk factor for cardiac surgery-related AKI, and a nationwide multicenter study supported the same conclusion (18). Our study also showed that men had a greater risk of developing FM-associated AKI than women. However, some large-scale observational studies on cardiac surgery identified female gender as an independent risk factor for AKI (19). Thus, this issue remains controversial and requires further investigation.

We also found that elevated baseline NT-pro BNP was a risk factor for FM-associated AKI, which was consistent with the results of Huang et al. (20). A possible mechanism is that elevated NT-pro BNP levels indicate severe cardiovascular dysfunction and impaired hemodynamics, which may promote the progression of AKI. Additionally, NT-pro BNP has also been confirmed as an independent predictor of postoperative AKI. Wang et al. (21) found that the inclusion of NT-pro BNP in their prediction model for AKI after cardiac surgery improved the model's predictive ability by 24%. Our study also showed that an elevated baseline procalcitonin level was a risk factor for FM-associated AKI. This finding is consistent with the results of a meta-analysis conducted by Feng et al. (22), which reported that procalcitonin may be a helpful predictor for AKI development. procalcitonin has been well recognized as an optimal biomarker for identifying infection (23), and its predictive ability for AKI has been widely studied in cardiovascular patients. Liu et al. (24) evaluated the association between AKI and serum procalcitonin levels in 328 patients with acute type A aortic dissection who received surgical treatment. Their results showed that patients with severe AKI had statistically higher serum procalcitonin levels at admission than other patients, and they concluded that serum procalcitonin is more accurate for predicting AKI than some traditional inflammatory biomarkers, such as C-reactive protein and white blood cell count. In addition, our study identiﬁed a reduced left ventricular ejection fraction as risk factor for FM-associated AKI. Left ventricular ejection fraction is a key indicator of the pumping ability of the heart. A decreased left ventricular ejection fraction indicates heart failure and circulation congestion, which results in reduced renal perfusion and an increased risk of AKI (25).

In our study, 66 patients (35.1%) who survived FM-associated AKI experienced progression to CKD. Multivariate logistic regression analysis showed that a prolonged interval from onset to treatment and elevated serum creatinine at discharge were independent risk factors for the progression of FM-associated AKI to CKD. The risk prediction model constructed using these indicators demonstrated excellent predictive accuracy for the progression of FM-associated AKI to CKD, aligning with models reported in previous studies (26, 27). When patients with FM-associated symptoms fail to receive timely and effective treatment, the duration of ischemia and hypoxia affecting the heart, kidneys, and other important organs is extended, leading to a gradual decrease in compensatory capacity. With the occurrence of serious damage to the kidneys, a continuous decline in cardiac output further aggravates renal ischemia, resulting in irreversible kidney damage and elevating the risk of CKD and ESRD. Our study also found that an elevated serum creatinine level at discharge was a risk factor for the progression of FM-associated AKI to CKD, which aligned with the results of previous reports (28–30). An elevated serum creatinine level at discharge indicates inadequate renal recovery post FM-associated AKI, which logically increases the risk of progression to CKD.

To date, several large-scale clinical retrospective studies have confirmed that AKI significantly increases the risks of CKD and ESRD. However, the specific mechanisms had not been fully clarified and mainly related to impaired repair and inhibition of the repair process after kidney injury (31, 32). Our study also found that although patients with AKI often regained normal renal function upon discharge, some patients experienced progression to CKD after 3 months. Therefore, it is advisable that FM patients with AKI be considered a high-risk group for CKD, and long-term close follow-up should be conducted after discharge to detect and block the progression from AKI to CKD.

Our study has the following strengths: the cohort was composed of exclusively FM patients, addressing a gap in clinical research regarding risk factors for FM-associated AKI and its progression to CKD. The study identified independent clinical predictors of FM-associated AKI and its progression to CKD, which can provide a preliminary basis for the early identification of high-risk patients and individualized clinical monitoring. Still, this study has some limitations: Firstly, this was a single-center retrospective cohort study, which has inherent biases such as selection bias and incomplete data, and therefore, the results need to be verified by multi-center prospective studies. Secondly, while a comprehensive list of clinical indicators were analyzed in this study, the clinical indicators cannot represent all risk factors for FM-associated AKI and its progression to CKD. New indicators (such as biomarkers of kidney injury, fibrosis markers, etc.) need to be discovered to further improve the predictive accuracy of models estimating the risk of progression from AKI to CKD. In addition, the study's followed-up period was restricted to 3 months, which is insufficient for a thorough and dynamic assessment of renal prognosis and progression to ESRD in patients with FM-associated AKI. A longer follow-up is needed to elucidate the long-term prognosis of this patient population.

## 5 Conclusions

In conclusion, our study demonstrated a high incidence of FM-associated AKI. The developed clinical risk prediction model can be helpful for identifying high-risk groups with poor renal prognosis from FM-associated AKI. However, studies with a larger sample size and longer follow-up period are still needed to determine the most appropriate clinical factors and biomarkers for the prediction of poor kidney prognosis in patients with FM-associated AKI.

## Data Availability

The raw data supporting the conclusions of this article will be made available by the authors, without undue reservation.
